# Primary Desmoplastic Melanoma of the Penis

**DOI:** 10.1155/2015/706572

**Published:** 2015-11-03

**Authors:** Julia T. Chu, Michael A. Liss, William W. Wu, Atreya Dash, Di Lu

**Affiliations:** ^1^Department of Pathology and Laboratory Medicine, University of California, Irvine, Orange, CA 92868, USA; ^2^Department of Urology, University of Texas Health Science Center San Antonio, San Antonio, TX 78229, USA; ^3^Department of Pathology, Weill Cornell Medical College and New York Presbyterian Hospital, New York, NY 10065, USA; ^4^Department of Urology, University of Washington, Seattle, WA 98195, USA

## Abstract

Desmoplastic melanomas are rare amelanotic melanomas that usually occur on skin with sun exposure. In this report, we present a 72-year-old man who presented with a desmoplastic melanoma of the penis. To our knowledge this represents the first reported case of primary desmoplastic melanoma of the penis. We discuss the pathologic differential and histologic evaluation.

## 1. Introduction

Penile cancers are relatively uncommon and histology other than squamous cell carcinoma is far less frequent. Desmoplastic melanomas usually occur on areas of skin exposed to sun. We report an unusual case of a 72-year-old man with primary desmoplastic melanoma of the penis, a site not often exposed to sunlight, which appears to be the first reported case. We also discuss salient points regarding the histologic diagnosis including the use of appropriate immunohistochemical staining.

## 2. Case Presentation

We present a 72-year-old Caucasian male with a history of neonatal circumcision, prostate adenocarcinoma having undergone radical prostatectomy in 1995, Hodgkin's lymphoma having received chemotherapy, and melanotic melanoma with resection of a lesion on the back in 2008 who presented to another institution with progressive urinary obstruction, urinary tract infections, and acute kidney injury. On clinical examination, the glans penis was firm with an obstructive penile lesion causing stenosis of the meatus. The meatus was biopsied at an outside hospital and a diagnosis of squamous cell carcinoma was made. The patient was then referred to UC Irvine for distal penectomy.

**Figure 1:**
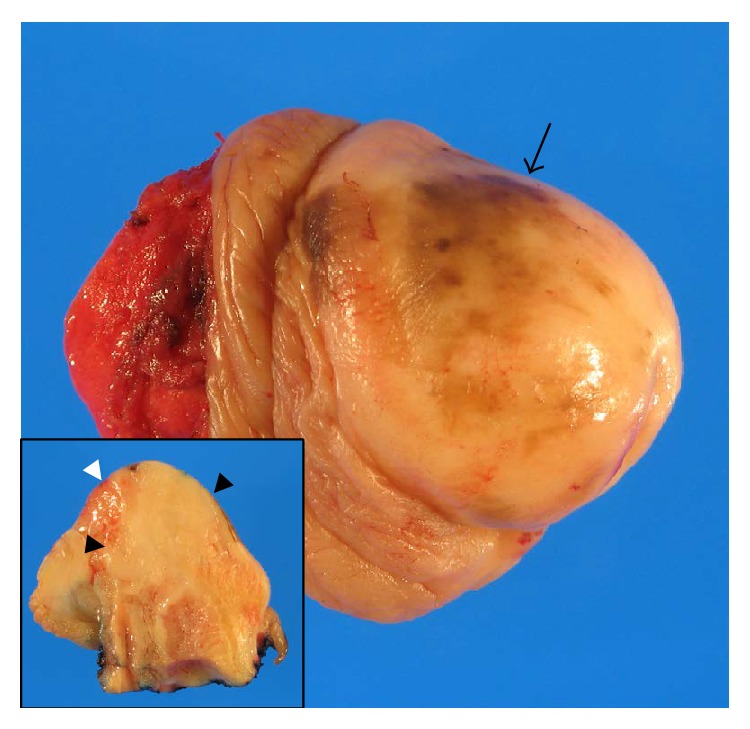
Glans of penis at penectomy. Gross examination shows scattered, flat, dark-brown discoloration mostly on the glans dorsal surface (black arrow) and ill-defined firmness of the entire glans. Cross sectioning (picture in picture) locates the firmness of glans to an indistinct, white, ulcer-free tumor measuring approximately 1.5 × 1.3 cm on cut surface. The tumor (black arrowheads) has invaded into and through the urethra (white arrowheads: urethral opening and margin; single black arrow: tumor invasion).

**Figure 2:**
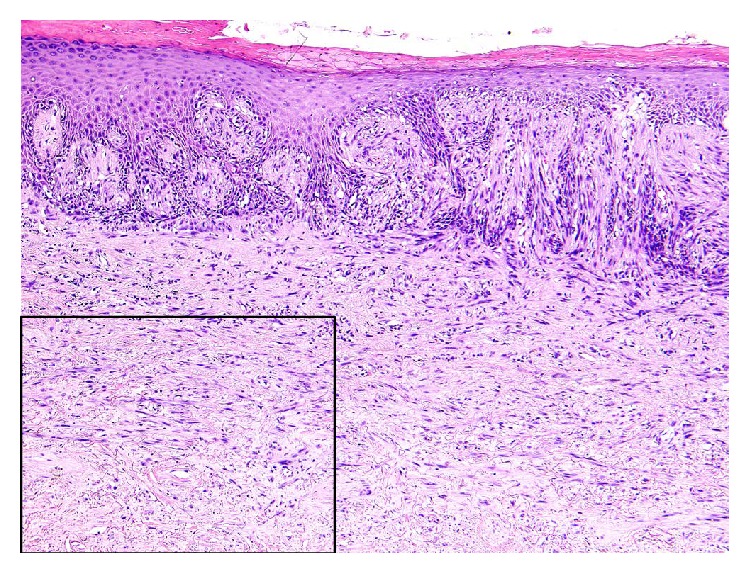
Histologic features of primary penile desmoplastic melanoma. Low-power photomicrograph of the penile glans depicts haphazardly arranged spindle tumor cells permeating in between collagen bundles and deeply infiltrating the dermis and corpus spongiosum within. In intimate association with the spindle tumor cells are focal atypical intraepidermal melanocytic proliferation and pseudoepitheliomatous hyperplasia in the overlying epidermis (left half of the H&E). S-100 is strongly positive in the spindle tumor cells (small photo).

Gross examination demonstrated a flat, dark-brown discoloration of the skin and ill-defined firmness of the entire glans. Cross sections revealed a poorly demarcated, white, firm tumor estimated to be 1.5 × 1.3 cm. The tumor had invaded into and through the urethra but without necrosis or ulceration (Figure 1). Histologically, disarrayed spindle tumor cells admixed in and between abundant (>90% of the entire neoplasm) collagen bundles and infiltrated the dermis and corpus spongiosum with conspicuous neurotropism. At higher magnification, moderate nuclear pleomorphism and rare atypical mitoses were present with evident neurotropism (Figure 2). In the epidermis overlying the spindle tumor cells were focal atypical melanocytic proliferation and pseudoepitheliomatous hyperplasia.

Histologic differential diagnosis included sarcomatoid squamous cell carcinoma, leiomyosarcoma, and desmoplastic melanoma. Immunohistochemically, the spindle tumor cells were strongly positive for S-100 protein and negative for AE1/AE3, HMB-45, melan-A, and smooth muscle actin (SMA). The atypical melanocytic cells at the epidermal-dermal junction were positive for HMB-45 and melan-A.

Immunohistochemical stains for desmin, epithelial membrane antigen (EMA), Cam 5.2, caldesmon, and muscle-specific actin antibody HHF-35 were all negative. BRAF V600 mutation was not detected by molecular polymerase chain reaction (PCR) method. A diagnosis of primary desmoplastic melanoma of the penis was made. An outside dermatopathology expert with published experience in desmoplastic melanoma concurred. Review of the previous biopsy showed similar morphology with pseudoepitheliomatous hyperplasia of the overlying skin.

## 3. Discussion

Desmoplastic melanoma, a rare type of amelanotic melanoma, primarily occurs on sun-exposed skin, such as the head and neck, trunk, and extremities, of elderly individuals with a predilection for males (M : F = 1.75 : 1) [1]. Although most lesions are amelanotic, 23% may be lightly pigmented and 6% heavily pigmented [2]. Desmoplastic melanomas are frequently associated with perineural invasion or neurotropism, resulting in deep infiltration and high local recurrence rate, but lower incidence of lymph node metastasis [3] with a rate of 6.7% [4]. Sentinel lymph node biopsy is not usually recommended [5]. Exceedingly rare cases can arise from the vulva [6] and penis [7]. To our knowledge, this is the first confirmed case of primary penile desmoplastic melanoma. Diagnostic confirmation by an immunohistochemistry panel composed of positive S-100, negative HMB-45, negative melan-A, negative AE1/AE3, and negative SMA is essential.

Sarcomatoid squamous cell carcinoma of the penis represents only 1-2%, up to 4% [8], of all penile carcinomas and is considered to be a high-grade, aggressive variant of squamous cell carcinoma with a mortality rate of up to 67%, recurrence rate of 12% [9], and inguinal lymph node metastasis in 89% of the patients [8]. Misdiagnosis of penile desmoplastic melanoma as the highly aggressive sarcomatoid squamous cell carcinoma would thus result in unnecessary standard or modified inguinal lymph node dissection in the absence of palpable inguinal adenopathy [10]. Immediate inguinal lymph node dissection can be curative for penile sarcomatoid squamous cell carcinoma but may result in severe morbidity and wound complications and chronic lymphedema in patients with misdiagnosed penile desmoplastic melanoma [11]. At 10 months after partial penectomy, our patient was alive and free of recurrent desmoplastic melanoma and inguinal lymphadenopathy.
